# Disrupted resting-sate brain network dynamics in children born extremely preterm

**DOI:** 10.1093/cercor/bhad101

**Published:** 2023-04-20

**Authors:** Nelly Padilla, Anira Escrichs, Elvira del Agua, Morten Kringelbach, Antonio Donaire, Gustavo Deco, Ulrika Åden

**Affiliations:** Department of Women’s and Children’s Health, Karolinska Institutet, Stockholm S- 171 76, Sweden; Computational Neuroscience Group, Center for Brain and Cognition, Department of Information and Communication Technologies, Universitat Pompeu Fabra, C/ de Ramon Trias Fargas, 25, 08018 Barcelona, España; Computational Neuroscience Group, Center for Brain and Cognition, Department of Information and Communication Technologies, Universitat Pompeu Fabra, C/ de Ramon Trias Fargas, 25, 08018 Barcelona, España; Department of Psychiatry, University of Oxford, Warneford Hospital, Warneford Ln, Oxford OX3 7JX, United Kingdom; Center for Music in the Brain, Aarhus University Hospital Nørrebrogade 44, Building 10G, 4th and 5th floor, 8000 Aarhus C, Denmark; Department of Neurology, Institute of Neuroscience, Hospital Clinic, Universidad de Barcelona and Institut D’investigacions Biomèdiques August Pi i Sunyer (IDIBAPS), 08036 Barcelona, Spain; Biomedical Research Networking Center in Bioengineering, Biomaterials and Nanomedicine (CIBER-BBN), Av. Monforte de Lemos, 3-5. Pabellón 11. Planta 0 28029 Madrid, Spain; Computational Neuroscience Group, Center for Brain and Cognition, Department of Information and Communication Technologies, Universitat Pompeu Fabra, C/ de Ramon Trias Fargas, 25, 08018 Barcelona, España; School of Psychological Sciences, Monash University, Melbourne, Clayton, VIC 3800, Australia; Institució Catalana de la Recerca i Estudis Avançats (ICREA), Passeig de Lluís Companys, 23, 08010, Barcelona, Catalonia, Spain; Department of Neuropsychology, Max Planck Institute for human Cognitive and Brain Sciences, Leipzig 04103, Germany; Department of Women’s and Children’s Health, Karolinska Institutet, Stockholm S- 171 76, Sweden; Department of Neonatology, Karolinska University Hospital, Stockholm S- 171 76, Sweden; Department of Biomedical and Clinical Sciences, Linköping University, Linköping, SE 58183, Sweden

**Keywords:** brain development, brain network dynamic, extreme prematurity, cognitive neurodevelopment

## Abstract

The developing brain has to adapt to environmental and intrinsic insults after extremely preterm (EPT) birth. Ongoing maturational processes maximize their fit to the environment and this can provide a substrate for neurodevelopmental failures. Resting-state functional magnetic resonance imaging was used to scan 33 children born EPT, at < 27 weeks of gestational age, and 26 full-term controls at 10 years of age. We studied the capability of a brain area to propagate neural information (intrinsic ignition) and its variability across time (node-metastability). This framework was computed for the dorsal attention network (DAN), frontoparietal, default-mode network (DMN), and the salience, limbic, visual, and somatosensory networks. The EPT group showed reduced intrinsic ignition in the DMN and DAN, compared with the controls, and reduced node-metastability in the DMN, DAN, and salience networks. Intrinsic ignition and node-metastability values correlated with cognitive performance at 12 years of age in both groups, but only survived in the term group after adjustment. Preterm birth disturbed the signatures of functional brain organization at rest in 3 core high-order networks: DMN, salience, and DAN. Identifying vulnerable resting-state networks after EPT birth may lead to interventions that aim to rebalance brain function.

## Introduction

Normal brain function involves a sequence of developmental events, such as cell division, migration, differentiation, axonal growth (Rakic and Lombroso 1998), network formation, and maturation (Kostovic and Jovanov-Milosevic 2006; Thomason 2020; Kostovic et al. 2021). Genes guide the first steps of brain development and the initial circuit architecture. Once the sensory systems have become responsive to environmental information, experience plays a fundamental role in forming and refining neural circuits (Uhlhaas et al. 2009; Berardi et al. 2015). This means that brain development and maturation are critically dependent on synchronized neural activity and activity-dependent plasticity (Uhlhaas et al. 2010; Benders et al. 2015). Appropriate stimuli during critical periods induce the events that are required to optimize the development of the structural connectivity that is required to support functional connectivity and its dynamics (Deco et al. 2014; Coronel et al. 2021). This framework implies that the brain is a complex system, which is characterized by a specific dynamical organization that is fundamental to supporting effective information processing and cognitive performance. It is based on the ability of brain regions to propagate local neural activity to the whole-brain network, which is known as intrinsic *ignition* (Deco and Kringelbach 2017), and the capacity to flexibly engage (integrate) and disengage (segregate) different brain areas throughout time (metastability) (Deco et al. 2017). The disruption of brain dynamics during development may lead to alternative developmental trajectories of the brain, where nonoptimal dynamics support brain disorders.

Being born extremely preterm (EPT), before 27 weeks of gestation, has been shown to disrupt the typical developmental trajectory of the brain. Compared with term-born children, children born EPT experience widespread alterations in gray and white matter at term-equivalent age (Padilla et al. 2015a) and during late childhood (Kvanta et al. 2021). These structural alterations have been related to both neonatal risk factors (Padilla et al. 2015a) and neurodevelopmental difficulties (Frie et al. 2016; Barnes et al. 2022), including autism spectrum disorders (Padilla et al. 2015b; Eklof et al. 2019). Overall, children who are born EPT have significantly different whole-brain structural (Fischi-Gomez et al. 2014) and functional organization (Padilla et al. 2020) to children born at term.

We have recently shown dynamic differences in the whole-brain network in children born EPT and typically developing children at 10 years of age (Padilla et al. 2020). In particular, in our previous study, we found that the EPT group showed reduced ignition and metastability across the whole-brain functional network compared with controls. Furthermore, using a whole-brain dynamical model that links the underlying structure with resting-state dynamics, we demonstrated that the EPT group presented significantly lower synchrony and reduced criticality than the control group. Finally, we found an altered hierarchy, predominantly in the rich-club areas, which drive information processing. However, it remains unknown which resting-state networks are affected in children born EPT or whether the disruptions of these networks are associated with cognitive function.

Here, we extend our previous study and apply a more fine-grained exploration of the underlying brain dynamics by studying the ignition and node-metastability within 7 well-known large-scale resting-state networks [i.e. dorsal attention network (DAN), frontoparietal, default-mode network (DMN), salience, limbic, visual, and somatosensory networks]. We hypothesized that children born EPT would have altered brain dynamics in high-order resting-state networks and that these changes would be correlated with their cognitive performance. Our aim was to investigate whether extreme prematurity would be related to changes in 2 dynamic brain aspects at the network level at 10 years of age. These were the capability of a brain area to propagate neural information, namely, intrinsic ignition, and whether this resulted in node-metastability, which is variations across time. We explored this by using resting-state functional magnetic resonance imaging (fMRI) and an intrinsic ignition network-based framework. In order to investigate the potential clinical relevance of these measures, we also aimed to explore the extent to which those brain dynamic measures were associated with cognitive function in EPT and term-born children at 12 years of age.

## Material and methods

### Population

This was a population-based regional cohort that focused on 111 EPT children born before 27 weeks of gestation in Stockholm County, Sweden, between 2004 and 2007. The children were invited for a follow-up MRI scan at 10 years ±2 months and 66 children born EPT and 46 term-born controls were enrolled. They were also invited to attend a developmental assessment at 12 years of age. The characteristics of the cohort have previously been described (Padilla et al. 2020). In brief, we excluded infants with congenital infections and malformations and those with severe brain lesions (periventricular leukomalacia or intraventricular hemorrhage (grades III–IV), focal brain lesions, cysts and malformations, persistent ventricular dilatation, or moderate or severe white matter abnormalities qualitatively defined by MRI examinations at term-equivalent age (Skiold et al. 2010). The term-born children were all healthy. Children were also excluded from both groups if uncorrectable motion artifacts were observed on their MRI scans. The final sample comprised 33 children born EPT and 26 term-born children (Supplementary Fig. 1).

### Cognitive assessment

Children underwent a cognitive assessment at 12 years of age using the Wechsler Intelligence Scale for Children—Fifth Edition (WISC-V). This provides scores for 5 indexes covering: fluid reasoning, working memory, processing speed, verbal comprehension, and visual spatial. These can be combined to provide a full-scale intelligence quotient (IQ).

### MRI data acquisition

The conventional MRI protocol that was used has previously been described (Padilla et al. 2020).

The children were asked to keep their eyes closed during the MRI scans, remain as motionless as possible, and not to fall sleep. In order to ascertain this, the families and the children were instructed about the process and were invited to participate actively in the process. We were not aware of any children that fell asleep during the procedure. Briefly, the MRI data were acquired using a Sigma HDx 3T MR scanner (GE Healthcare, Illinois, USA). The MRI protocol included a sagittal 3D-T1 weighted with a BRAVO SPGR sequence: time to inversion = 400 ms, field of view = 240 × 240 mm^2^, flip angle = 12° voxel size 1 × 0.938 × 0.938 mm^3^, and slice thickness = 1.0 mm. The resting-state fMRI data were acquired with a gradient-echo EPI sequence, with a total of 300 volumes: time repetition/time echo = 2,000/30 ms; flip angle = 70° voxel size 3.0 × 3.0 × 3.5 mm^3^ with full-brain coverage.

### fMRI preprocessing and motion censoring

The resting state fMRI data set was preprocessed using FSL software, version 5.0.5 (FMRIB Software Library, Oxford, UK). All the raw resting state fMRI data and outputs from each preprocessing step were visually examined. Extreme motion data were eliminated from the start of the process. The initial steps were slice-timing correction, volume realignment, co-registration of the functional image to the T1-weighted image, and registration from high-resolution structural data to the study-specific template. We used aggressive ICA-AROMA, which provides ICA-based automatic removal of motion artifacts to identify and remove residual-motion-related artifacts (Pruim et al. 2015). We calculated for each participant: outlier volumes and root mean square frame displacement using the FSL motion outliers’ tool (FMRIB) (Dipasquale et al. 2017); DVARS, where D was the temporal derivative of time courses and VARS referred to the root mean square variance over voxels; and the number of outliers (>75th percentile+1.5 interquartile range). We also used a frame displacement threshold of 0.2 mm (Power et al. 2015). fMRI series that had more than 15% of outlier volumes in the frame displacement or DVARS with more than 0.2 mm of motion displacement were excluded from the study (Supplementary Table 1). The time courses were used to extract 7 resting-state networks, using a resting-state atlas of the human brain’s functional connectivity (Schaefer et al. 2018).

**Fig. 1:**
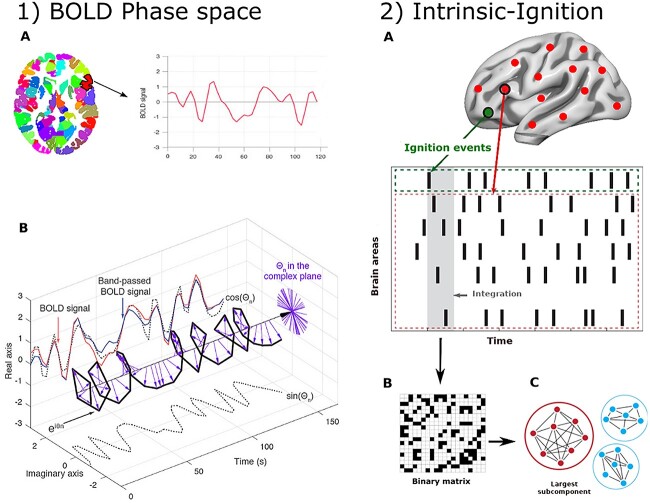
Intrinsic ignition framework. 1) We extracted the BOLD time series and computed the phase space of the BOLD signal for each of the 100 brain areas. 1A) the time series for each brain area was extracted using a resting-state atlas (Schaefer et al. 2018), and 1B) then, the phase space of the BOLD signals for each brain area was assessed by calculating the Hilbert transform. The BOLD signal (red) was filtered between 0.01 and 0.07 Hz (blue) and converted with the Hilbert transform into an analytical signal, represented by its instantaneous amplitude and its phase $\varphi$ (with real and imaginary components). The phase dynamics are represented in the complex plane as ${e}^{i{\varphi}_n}$ (black bold line), with $\sin \varphi$representing the imaginary part (black dotted lines) and $\cos \varphi$representing the real part. The purple arrows denote the Hilbert phases for a given brain area over time. 2) Intrinsic ignition. 2A) Events were obtained using a threshold (Tagliazucchi et al. 2012) (green area), and the activity in the rest of the network was calculated for each ignition event crossing the threshold (red stippled area), in the 4TR time window (gray area). 2B) A binarized phase-lock matrix was obtained from the time window. 2C) The integration was obtained from this phase-lock matrix by calculating the largest subcomponent (Deco et al. 2017). Finally, we repeated the process for each driving event and the framework returned the ignition and node-metastability for each brain area across the network. Figure adapted from 2 studies (Deco and Kringelbach 2017; Escrichs et al. 2019).

### Intrinsic ignition framework

Intrinsic ignition is a local and global measure of the brain’s dynamical complexity (Deco and Kringelbach 2017) and has been used in multiple resting-state fMRI studies (Escrichs et al. 2019, 2021; Padilla et al. 2020; Alonso et al. 2020a; Uribe et al. 2021). This framework assesses the effect of naturally activated events, which reflect a brain region’s capacity to propagate neural activity to other regions. Briefly, the Blood Oxygenation Level Dependent (BOLD) time series were transformed to phase space, by filtering the signals within the narrowband (0.01–0.07 Hz), and the Hilbert transform was computed to obtain the signal phases between each pair of brain regions at each time point (Fig. 1). Ignition events were defined as a binary signal, by transforming the time series into *z*-scores $z_i(t)$ and fixing a threshold, *θ*, so that the binary sequence $\sigma(t)=1\ if\ z_i(t)\gt \theta$ was crossing the threshold from below and ${\sigma} (t)=0$ otherwise (Fig. 1B) (Tagliazucchi et al. 2012; Deco et al. 2017). Then, a phase-lock matrix $P_jk(t)$ was calculated, describing the state of phase synchronization between brain regions *j* and *k* at time *t* as: 


$$ P_jk(t)=\text{e}^{-3\mid{\varphi}_{j}(\text{t}){\varphi}_{k}(\text{t})}$$

where ${\varphi}_{j}(\text{t})$ and ${\varphi}_{k}(\text{t})$ correspond to the phases of the BOLD time series for the brain regions at time *t*. Then, the integration was defined by estimating the length of the largest connected component in the phase-lock matrix and the integration value was computed as the length of the connected component (the largest subcomponent). The framework returned the mean integration (ignition) and the standard deviation (node-metastability) across the network. The ignition represents the spatial diversity across the network, whereas the node-metastability represented the variability over time for each brain region. This framework was computed independently for 7 resting-state networks from the Schaefer et al. (2018) parcellations atlas: the salience, dorsal attention, frontoparietal, default mode, limbic, visual, and somatosensory networks.

### Statistics

Characteristics of the groups and cognitive variables were tested for normality and homogeneity before each analysis. The data were analyzed using SPSS, version 20 (IBM Corp, New York, USA). Independent samples *t*-test and Mann–Whitney U tests were applied, where appropriate, to assess group differences, and a 2-sided *P*-value of *P* < 0.05 was considered statistically significant. To test the differences between groups in ignition and node-metastability across resting-state networks, we used a Montecarlo Permutation test (50,000 permutations). Spearman’s correlation was used to assess correlation between the brain data in the networks that were significantly different between groups at 10 years of age and the developmental scores at 12 years of age. All *P*-values (within each network and each group) were corrected for multiple comparisons following the false discovery rate method (FDR) (Hochberg and Benjamini 1990). Given the homogeneity of each group, any clinical covariate was incorporated in this analysis. We also applied the FDR method to correct for multiple comparisons when testing the differences between groups in the 7 resting-state networks and assess the correlations between brain measures and developmental scores.

## Results

### Demographics

The characteristics of the children included in this study have previously been described (Padilla et al. 2020). Table 1 shows the perinatal data at 10 years of age and the WISC-V scores at 12 years of age. From the original cohort of 112 children (66 EPT and 46 term children), 44 children (26 EPT and 18 term) were excluded because of low-quality MRI studies (Supplementary Fig. 1). In all, 26 children were excluded because of motion (14 excluded according to the number of outliers and FD, 4 had extreme motion, 7 fMRI corrupted, and 1 child had braces) (Supplementary Fig. 1). The drop-out analyses of the children born EPT that were not included in the study, because of low structural and/or fMRI quality, compared with those included in the study, did not show significant differences in terms of neonatal characteristics, age at MRI or at developmental assessment (Supplementary Tables 1 and 2). The 2 groups did not differ in terms of motion parameters (Supplementary Table 3).

**Table 1:** Characteristics of the groups.

Characteristic	EPT	Term	Statistic (*P*)
	*n* = 33	*n* = 26	
Perinatal data			
Gestational age (weeks)	25.70 (0.95)	39.93 (1.12)	*t* −52.8 (<0.001)
range	23.5–26.6	37.3–41.5	
Birth weight (g)	856.2 (173.4)	3.663 (421.0)	*t* −31.8 (<0.001)
range	550–1161	2.875–4.100	
Gender (boy/girl)	13/20	14/12	Fisher’s test (0.17)
Age at MRI (years)	10.06 (0.82)	9.92 (0.88)	*t* 0.65 (0.52)
range	9.0–11.5	8.0–11.8	
Age at WISC	11.8 (0.40)	11.6 (4.07)	0.30
WISC index	EPT *n* = 29	Term *n* = 21	Statistic *(p)*
	mean (SD)	mean (SD)	
Verbal comprehension	100.70 (16.14)	118.76 (16.45)	*t* −3.71 (<0.001)
Visuospatial	91.50/14.97)	102.57 (14.60)	*t* −2.50 (0.016)
Fluid reasoning	94.79 (11.08)	106.85 (10.48)	*t* −3.73 (<0.001)
Working memory	90.12 (17.61)	101.38 (12.42)	*t* −2.44 (0.019)
Processing speed	95 (15.99)	107.52 (13.66)	*t* −2.80 (0.008)
Total IQ	94.29 (14.11)	112.42 (12.70)	*t* −4.50 (<0.001)

EPT, extremely preterm group; SD, standard deviation; WISC-V, Wechsler Intelligence Scale for Children—Fifth Edition V; IQ, intelligence quotient.

### Cognitive assessments at 12 years of age

Developmental assessments were carried out on 29 children born EPT children and 21 term controls at 12 years of age. There were significant differences in the cognitive scores between the 2 groups (Table 1). Overall, the EPT group had lower scores in all cognitive indexes than the full-term group. All the *P*-values survived correction for multiple comparisons using the FDR.

### Results of the fMRI scans at 10 years of age

**Fig. 2:**
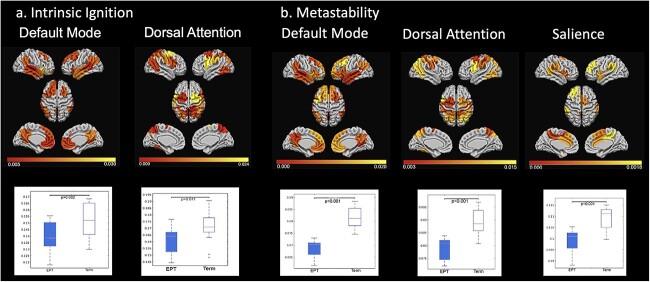
a) Ignition measure. The EPT group exhibited significantly lower ignition values in the DMN and DAN than the control group. Rendered brains showed the absolute difference between the groups, with the regions in yellow representing the largest differences. The boxplots show the ignition values for each group and resting-state network. b) Node-metastability measure. The EPT group exhibited lower values of node-metastability in the DMN, DAN, and salience networks than the control group. *P*-values are based on the Wilcoxon rank-sum test.

We carried out fMRI scans on 34 children born EPT and 26 children born at term. When they were compared with the term group, the children born EPT showed significantly lower mean ignition and node-metastability in the DMN (*P* = 0.002 and *P* = 0.00001, respectively) and DAN (*P* = 0.011 and *P* = 0.00001, respectively). Node-metastability was also reduced in the salience network in the EPT group (*P* = 0.02) (Fig. 2). Supplementary Fig. 2 displays the results for the 7 networks. Furthermore, we have applied a bootstrap approach (Efron and Tibshirani 1994) to assess the significance of the results. We found statistical significance in the DMN and a consistent pattern for salience and DAN networks (Supplementary Fig. 3). In addition, in the EPT group, the intrinsic ignition values in the salience network were negatively correlated with cognitive performance in the fluid index (*r* = −0.404, *P* = 0.030), visuospatial index (*r* = −0.388, *P* = 0.038), and total IQ (*r* = −0.428, *P* = 0.020). In the term-born children, the node-metastability values in the DAN were positively correlated with cognitive performance in the processing speed assessments (*r* = 0.492, *P* = 0,023), visuospatial index (*r* = 0.500, *P* = 0.021), and total IQ (*r* = 0.563, *P* = 0.008). The correlations in the EPT group did not survive the FDR corrections (*r* = 0.076) for all significant correlations (Table 2), but the data for the term group did.

## Discussion

This study used an intrinsic ignition network-based framework to assess whether the integration (intrinsic ignition) elicited by specific resting-state networks, and the variability across time of brain nodes in these networks (node-metastability), differed between children born EPT and at term. We also examined whether those measures were associated with cognitive performance. There were 3 major findings. First, EPT birth was related to reduced intrinsic ignition in the DMN and DAN. Second, extreme prematurity was also related to reduced node-metastability in the DMN, DAN, and salience network. Third, the intrinsic ignition and node-metastability values were correlated with cognitive performance in both groups, but only survived in the term group after adjustment. Taken together, these findings suggest that functional brain dynamics are compromised when an infant is born EPT, even during late childhood, and that 3 key higher-order networks for cognitive processes, the DMN, DAN, and salience network, are involved. This study also offers new theoretical knowledge that can improve our understanding of the anatomical bases of functional impairments reported in children born EPT.

### EPT birth and ignition and node-metastability in the DMN, salience network, and DAN

This study showed that the DMN, the functional dynamic patterns of the salience network, and the DAN, which is part of the executive network, were all affected by EPT birth and showed reduced intrinsic ignition and reduced node-metastability. This finding is important given that these 3 networks have been defined as the so-called unifying triple network model, which is disrupted in several psychiatric and neurological disorders (Menon 2011, 2019). Particularly during childhood and adolescence, these networks play a crucial role in neurodevelopment (Uddin et al. 2011). Our results align with previous studies showing that the functional connectivity in the triple networks is altered in stress, depression, or trauma exposure in adolescents and early life adversity (Zeev-Wolf et al. 2019; Fadel et al. 2021; Macedo et al. 2022). Overall, the present study shows for the first time that being born EPT impacts the functional connectivity of the DMN, DAN, and salience networks and thus provides important insights into neurodevelopmental impairments related to extreme prematurity in late childhood in the triple network model.

Studies have shown that the DMN is more likely to be activated during internally directed activities (Raichle 2015; Weber et al. 2022) and the DAN is activated during cognitive goal-directed tasks. The salience network represents the homeostatic system (Seeley et al. 2007) and provides the core orchestration of brain dynamics, maintaining the balance of the internally and externally directed systems (Menon and Uddin 2010). Reduced ignition in the DMN and DAN suggests imbalances in their capacity to integrate information over time (Deco and Kringelbach 2017), with the reduced node-metastability in the 3 networks, this suggests an imbalance in the orchestration of brain dynamics and highlights the key role played by the salience networks in switching between the internal (DMN) and external demands (DAN).

Reduced ignition in the EPT group, involving the DMN and the DAN, may reflect a delayed pattern of maturation. This may be the result of 2 factors, namely, anatomical and neurodevelopmental consequences because of prematurity. Immature neurons and networks display abnormal, long-lasting immature properties (Ackman et al. 2009). In children born EPT, disturbances in the maturation of cortical networks are likely to reflect disturbed development of the structure (Padilla et al. 2015a; Kvanta et al. 2021), function (Fransson et al. 2007), and dynamic properties of the brain (Padilla et al. 2020). These may compromise the capability of the brain areas to propagate neural activity (Hagmann et al. 2010).

**Table 2:** Correlations between brain data and cognitive performance at 12 years of age.

	EPT *n* = 29		Term *n* = 21	
Network/WISC index	Ignition *r* (*P*)	Metastability *r* (*P*)	Ignition *r* (*P*)	Metastability *r* (*P*)
DMN				
Processing speed	0.18 (0.40)	0.001 (0.99)	−0.12 (0.58)	−0.12 (0.59)
Fluid index	−0.17 (0.42)	0.004 (0.98)	−0.33 (0.14)	−0.08 (0.70)
Visuospatial	−0.28 (0.17)	−0.03 (0.87)	−0.25 (0.26)	−0.01 (0.94)
Working memory	0.07 (0.71)	0.03 (0.85)	−0.29 (0.19)	−0.01 (0.95)
Verbal index	−0.04 (0.83)	0.05 (0.78)	−0.11 (0.61)	0.05 (0.80)
Total IQ	−0.08 (0.70)	−0.05 (0.81)	−0.26 (0.24)	0.02 (0.92)
Dorsal attention				
Processing speed	0.14 (0.50)	−0.12 (0.54)	0.02 (0.92)	**0.49 (0.02)** ^**a**^
Fluid index	−0.29 (0.16)	−0.008 (0.97)	0.16 (0.48)	0.44 (0.04)
Visuospatial	−0.14 (0.49)	0.003 (0.90)	0.09 (0.67)	**0.50 (0.02)** ^**a**^
Working memory	−0.05 (0.80)	−0.19 (0.36)	0.01 (0.93)	0.32 (0.15)
Verbal index	−0.03 (0.86)	−0.17 (0.40)	0.30 (0.17)	0.29 (0.19)
Total IQ	0.06 (0.78)	0.24 (0.16)	0.26 (0.24)	**0.56 (0.008)** ^**a**^
Salience				
Processing speed	−0.28 (0.14)	−0.18 (0.38)	0.41 (0.05)	0.32 (0.15)
Fluid index	−040 (0.03)^a^	−0.21 (0.17)	0.27 (0.33)	0.04 (0.84)
Visuospatial	−0.38 (0.04)^a^	−0.31 (0.13)	0.27 (0.22)	0.27 (0.22)
Working memory	−0.27 (0.14)	−0.30 (0.15)	0.07 (0.75)	−0.008 (0.97)
Verbal index	−0.16 (0.40)	0.09 (0.66)	0.22 (0.33)	−0.05 (0.81)
Total IQ	−0.42 (0.02)^a^	−0.20 (0.33)	0.35 (0.11)	0.12 (0.58)

EPT, extremely preterm; DMN, default-mode network; IQ, intelligence quotient; *r*, Spearman’s rho. ^a^*P*-values < 0.05. Bold values remained significant after correction for multiple comparisons.

The EPT group also showed reduced node-metastability in the DMN, salience network, and DAN. In line with previous studies, we found the node-metastability to be a more sensitive measure than the ignition (Escrichs et al. 2019, 2021; Alonso et al. 2020b). This is because node-metastability captures the functional variability of each brain area over time, describing its versatility (i.e. how each area fluctuates across time), whereas the ignition captures its spatial diversity (Escrichs et al. 2019, 2021; Alonso et al. 2020a). Metastability helps to explain how neural networks coordinate their activity, namely, network switching, to support cortical function. The ability of the cortical networks to switch between different patterns of connectivity increases during development and is a hallmark of maturity (Uhlhaas et al. 2009). This is consistent with maturational changes in the myelination of connections, neurotransmitters (Uhlhaas et al. 2010), and excitation and inhibition balance (Sydnor et al. 2021). The insula is one of the most densely connecting hubs in the salience network and is a major source of the transient bursting events that are critical for brain maturation in preterm infants (Arichi et al. 2017). This maturational pattern seems to be delayed in children born EPT, who show reduced metastability when they are compared with term children. Many of the nodes within the salience network and DMN mature before birth. Being born EPT is likely to induce disturbances in their structural and functional development (Lordier et al. 2019; Lammertink et al. 2020) and their activity patterns and interactions (Chai et al. 2017). These may impair salience processing (White et al. 2014; Lordier et al. 2019). These specific maturational patterns could point to a dynamical brain disorder, linked to EPT birth, which is characterized by reduced intrinsic ignition and node-metastability in 3 core neurocognitive networks: the DMN, DAN, and salience network. Identifying vulnerable resting-state networks in children born EPT may allow us to design interventions that aim to rebalance their brain function. Mathematical whole-brain models that simulate resting-state networks and whole-brain dynamics have advanced our understanding of the structure and function relationship in the brain and the potential repercussions of disrupted connectivity from injury or disease. Such whole-brain models combined with in silico (artificial) simulations have opened the possibility of discovering potential stimulation targets to shift patients’ disrupted brain dynamics toward more healthy states (Deco et al. 2014, 2019; Escrichs et al. 2021). Consequently, such a theoretical approach can help us to develop studies in vivo using noninvasive stimulation techniques, i.e. transcranial magnetic stimulation or transcranial alternating current stimulation, to investigate future therapeutical applications in brain disorders.

### Cognitive performance, ignition, and node-metastability correlations

This study has demonstrated correlations between brain dynamic measures at 10 years of age, namely, intrinsic ignition and node-metastability, and cognitive performance in several domains and total IQ at 12 years of age. Previous adult studies have suggested critical links between these brain dynamic measures and cognition (Hellyer et al. 2015), attention-regulation, fluidity (Uribe et al. 2021), and even symptoms associated with depression (Alonso et al. 2020b). In the term group, we found significant correlations between brain measures in the salience and DAN and cognitive performance on 2 main indexes assessed: visuospatial and processing speed indexes. Those 2 indexes could be considered as a condition for broader neurodevelopment in the context of focus attention (Beunders et al. 2021). Despite the lack of comparable studies, our results were consistent with a previous study that reported correlations between metastability values, processing speed, and visuospatial abilities in adults (Hellyer et al. 2015; Alderson et al. 2020). This relationship could be explained because of the need of rapid switching between competing task demands. In the EPT group, the salience network showed negative correlations with fluid reasoning index, which is the cornerstone of the human condition (Ferrer et al. 2009), and with the visuospatial index. We cannot give too much credence to these findings, because the correlations observed in the EPT group were no longer significant once the data had been corrected for multiple comparisons. The absence of a similar result in EPT children compared with the term group could suggest developmental differences between EPT children and typically developing children, beyond just a cognitive deficit. More studies are needed to prospectively evaluate how brain dynamic measures during late childhood are associated with later cognitive outcomes, in children born EPT.

### Strengths and limitations

The strengths of this study include the well-defined cohort of children born EPT at up to 27 weeks of gestation and followed up at 10 and 12 years of age. A possible limitation of this study was the number of children who could not be included in the MRI analysis because of rigorous entry and data quality criteria. However, the drop-out analyses showed that outcomes were not different between the children included in the study and those excluded because of low-MRI quality. Thus, the sample of children included was representative of the whole population. Additionally, we did not have socioeconomic status data for all of the children, and, therefore, we could not evaluate the influence of this variable on cognitive outcomes. Studies with larger samples would help to determine the significance of the changes found in the EPT group.

In conclusion, reduced ignition and node-metastability were demonstrated in the EPT group at 10 years of age and these involved 3 core networks: the DMN, DAN, and salience networks. These processes also correlated with cognitive performance at 12 years of age in both groups, but only survived in the term group after adjustment. Our findings are a critical first step toward using dynamical biomarkers, to predict current biological risks or identify early signs of developmental disorders. This study has important implications for our ability to understand and treat cognitive difficulties in children born EPT. Identifying vulnerable resting-state networks in children born EPT may allow us to design interventions that aim to rebalance their brain function.

## Supplementary Material

Supplementary_Material_bhad101

## Data Availability

The data will be shared on reasonable request to the corresponding author. Additional data can be requested from fouu@sll.se - a non-author institutional point of contact at Karolinska University Hospital.
