# Supplementary material for: Disrupted resting-sate brain network dynamics in children born extremely preterm
Source: Cereb Cortex. 2023 Apr 20;33(13):8101–9. doi: 10.1093/cercor/bhad101 (PMC10321088; doi:10.1093/cercor/bhad101)
Supplement: Supplementary_Material_bhad101 [file supplementary_material_bhad101.pdf]

Supplementary Material

Figure 1. Population

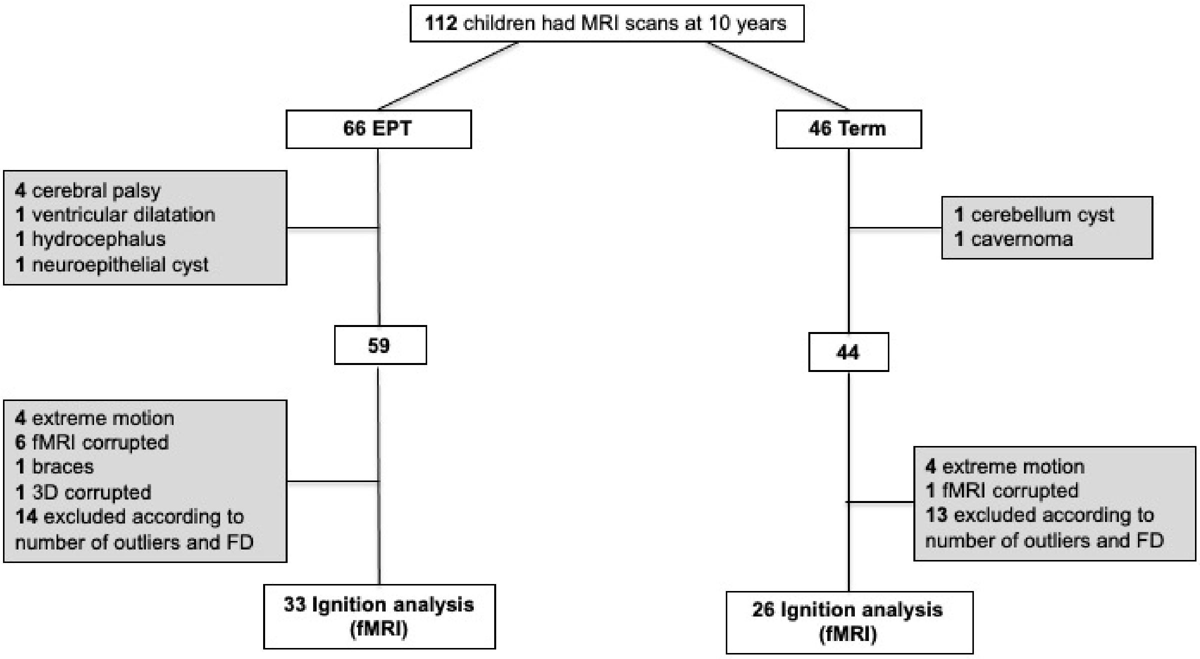

EPT, extremely preterm; fMRI, functional magnetic resonance imaging; FD, frame displacement. Number of outliers using FSL motion-outliers-tool (>75thpercentile + 1.5 Inter-Quartile Range) and FD 0.2 mm (Power et al., 2015)

Supplementary Figure 2. Ignition and metastability across resting-state networks between EPT and term groups

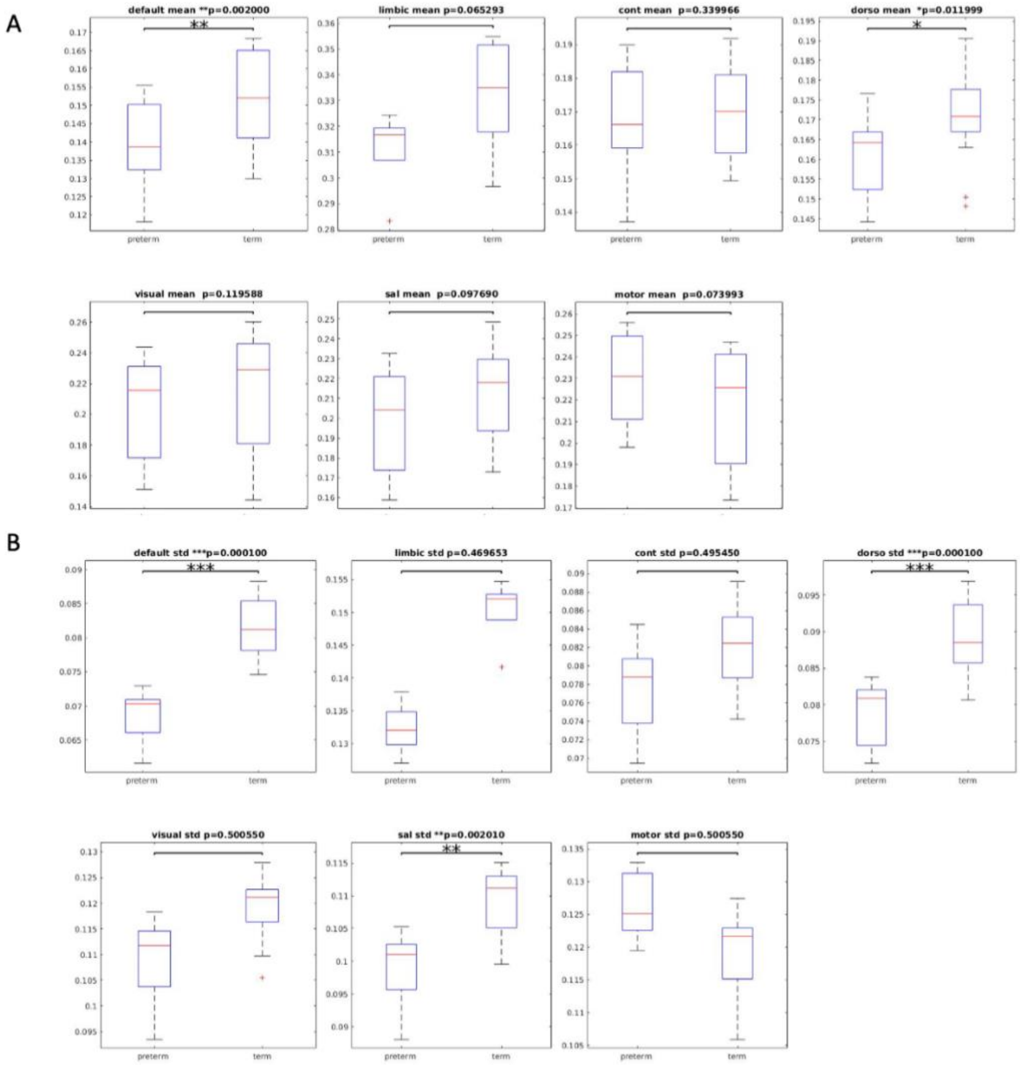

A. Ignition. B. Metastability. Networks from left to right, 1st row:default mode, limbic, executive, dorsal attention. 2nd row: visual, salience, motor. EPT, extremely preterm.

P-values were assessed using the Monte Carlo permutation test and corrected for multiple comparisons for each measure using FDR, *P < 0.05, **P < 0.01 and ***P < 0.001.

Supplementary Figure 3.

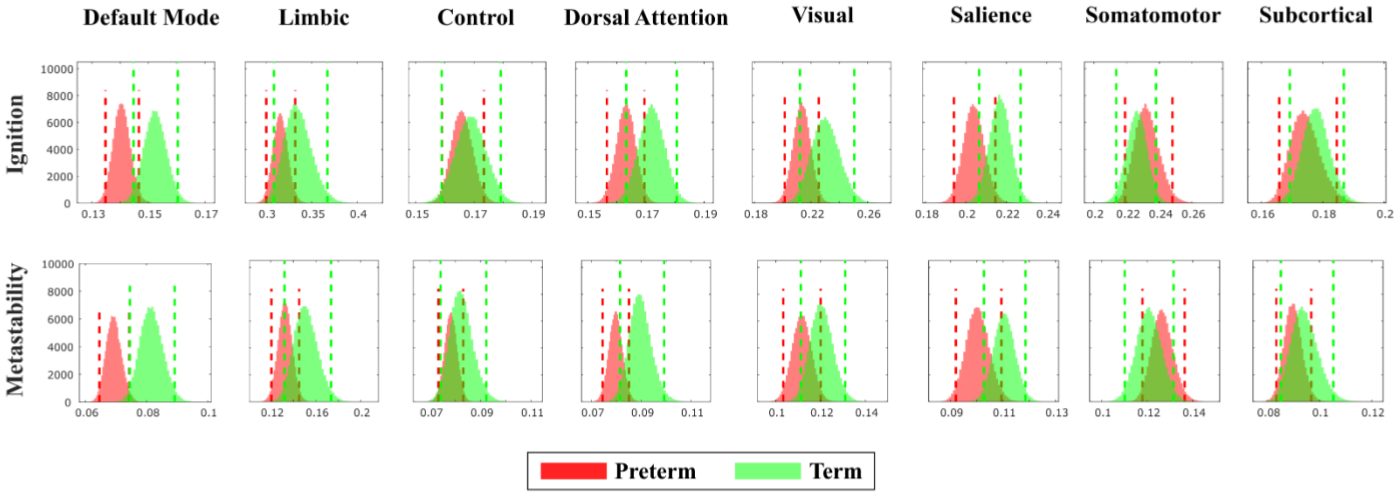

Supplementary Figure 3: We applied a 100,000-sample bootstrap approach (Efron & Tibshirani, 1994) to brain measures. For each bootstrap sample, we obtained the ignition and node-metastability and saved the median values for each group (plotted in the histograms). The dashed lines mark the 95% confidence intervals. We confirm the statistical significance of the DMN and a consistent pattern for salience and DAN networks.

Supplementary Table 1. Drop-out analysis for children born EPT with and without high quality MRI

**Neonatal characteristic**	**EPT included**	**EPT not included**	**Statistic (*p* value)**
	**N=33**	**N=33**	
Gestational age at birth, months (SD)	25.7 (0.95)	25.3 (1.0)	t=1.38 (0.17)
Weight at birth, g (SD)	0.86 (0.17)	0.81 (0.12)	t= 1.11 (0.26)
Antenatal steroids, n (%)	29/32 (90.6)	30/31 (96.8)	FET, 0.61
Apgar score at 5 minutes, mode (range)	8.13 (1.49)	7.81 (1.75)	t=0.77 (0.74)
Small for gestational age*	2/33 (6.1)	2/32 (6.3)	FET, 1.00
Clinical Risk Index for Babies score, mean ± SD	5.0 ± 3.7	5.13 ± 2.9	t=-0.04 (0.96)
Days on ventilator, mean ± SD	10 (13.08)	12.90 (12.2)	-0.90 (0.37)
Postnatal corticosteroid therapy, n (%)	4/32 (12.5)	5/31 (16.1)	FET, 0.73
Bronchopulmonary dysplasia,** n (%)	9/30 (30.0)	15/31 (48.4)	FET, 0.19
Sepsis (clinical diagnosis), n (%)	22/32 (68.7)	22/31 (71)	FET 1.00
Necrotizing enterocolitis, n (%)	2/32 (6.3)	6/31 (19.4)	FET, 0.14
Patent ductus arteriosus treated with surgery, n (%)	8/32 (25.0)	11/31 (25.5)	FET, 0.41
Patent ductus arteriosus treated with ibuprofen, n (%)	19/32 (59.4)	22/29 (75.9)	FET, 0.18
Low-grade intraventricular hemorrhage, n (%)	9/30 (30.0)	12/27 (44.4)	FET, 0.28
Mild white matter abnormalities (score 7-9), n (%)	12/29 (41.4)	15/26 (57.7)	FET, 0.28
Retinopathy of prematurity, n (%)	23/29 (79.3)	25/30 (83.3)	FET, 0.74
**Characteristics at late childhood**			
Age at MRI	10.06 (0.82)	9.84 (0.83)	t=1.43 (0.15)
Age at developmental assessment	12.12 (0.23)	12.22 (0.29)	t=-1.43 (0.15)
EPT, extremely preterm; SD, standard deviation; *Oxygen at 36 weeks of gestation. **Small for gestational age, < -2SD, FTE, Fisher's Exact Test.			

Supplementary Table 2. Drop-out analysis for children born EPT with and without developmental assessment at 12 years of age.

**Neonatal characteristic**	**EPT included**	**EPT not included**	**Statistic (*p* value)**
	N=29	N=26	
Gestational age at birth, months (SD)	25.63 (0.97)	25.52 (0.99)	t=0.40 (0.68)
Weight at birth, g (SD)	0.84 (0.17)	0.83 (0.12)	t= 0.29 (0.76)
Antenatal steroids, n (%)	25/28 (89.3)	24/25 (96.0)	FET, 0.61
Apgar score at 5 minutes, mode (range)	8.18 (1.54)	8.08 (1.47)	t=0.23 (0.81)
Small for gestational age*	2/33 (6.1)	2/32 (6.3)	FET, 1.00
Clinical Risk Index for Babies score, mean ± SD	5.0 ± 3.64	5.04 ± 2.85	t=-0.04 (0.96)
Days on ventilator, mean ± SD	9.86 (13.10)	12.25 (12.0)	-0.68 (0.49)
Postnatal corticosteroid therapy, n (%)	3/28 (10.7)	3/25 (12.0)	FET, 1.00
Bronchopulmonary dysplasia,** n (%)	8/27 (29.6)	14/25 (56.0)	FET, 0.09
Sepsis (clinical diagnosis), n (%)	19/28 (67.9)	17/25 (68.0)	FET 1.00
Necrotizing enterocolitis, n (%)	2/28 (7.1)	5/25 (20.0)	FET, 0.23
Patent ductus arteriosus treated with surgery, n (%)	8/28 (28.6)	7/25 (28.0)	FET, 1.00
Patent ductus arteriosus treated with ibuprofen, n (%)	16/28 (57.1)	16/25 (64.0)	FET, 0.77
Low-grade intraventricular hemorrhage, n (%)	10/28 (35.7)	14/25 (56.0)	FET, 0.17
Mild white matter abnormalities (score 7-9), n (%)	16/26 (38.5)	10/20 (21.7)	FET, 0.55
Retinopathy of prematurity, n (%)	23/29 (79.3)	25/30 (83.3)	FET, 0.74
**Characteristics at late childhood**			
Age at MRI	10.06 (0.84)	9.88 (0.84)	t=-0.66 (0.44)
Age at developmental assessment	12.12 (0.23)	12.17 (0.24)	t=-0.76 (0.45)
EPT, extremely preterm; SD, standard deviation; *Oxygen at 36 weeks of gestation. **Small for gestational age, < -2SD, FTE, Fisher's Exact Test.			

Supplementary Table 3. Motion estimation after AROMA

**Parameter**	**Preterm included** N=33	**Term** N=26	**Statistic, (p value)**
Average FD-RMS motion estimation, mean SD)	0.10 (0.05)	0.08 (0.04)	*t* 1.44 (0.15)
Average DVARS-RMS estimation, mean SD)	5.97 (0.84)	5.84 (0.91)	*t* 0.23 (0.81)
Number of frame outliers, metric FD-MRS	19.9 (10.12)	19.52 (9.5)	*t* 0.14 (0.88)
Number of frame outliers, metric DVARS	14.4 (6.96)	14.4 (9.14)	*t* 0.02 (0.98)
Rotation X	0.11 (1.78)	0.45 (1.61)	*t* -0.73 (0.46)
Rotation Y	-0.26 (0.60)	0.13 (0.54)	*t* -1.03 (0.30)
Rotation Z	0.25 (1.53)	-0.57 (1.69)	*t* 1.87 (0.06)
Translation X	-0.03 (0.19)	0.02 (0.12)	*t* -1.29 (0.20)
Translation Y	-0.10 (0.48)	0.003 (0.13)	*t* -1.09 (0.27)
Translation Z	-0.007 (0.22)	0.009 (0.13)	*t* -0.31 (0.75)

FD, frame displacement; RMS, root mean square; DVARS, D is temporal derivative of time courses and VARS is RMS variance over voxels.
